# Physical Activity With Eduball Stimulates Graphomotor Skills in Primary School Students

**DOI:** 10.3389/fpsyg.2021.614138

**Published:** 2021-03-04

**Authors:** Sara Wawrzyniak, Ireneusz Cichy, Ana Rita Matias, Damian Pawlik, Agnieszka Kruszwicka, Michal Klichowski, Andrzej Rokita

**Affiliations:** ^1^Department of Team Sports Games, University School of Physical Education in Wroclaw, Wroclaw, Poland; ^2^Department of Sports and Health, University of Évora, Évora, Portugal; ^3^Department of Biology and Motor Sports Fundamentals, University School of Physical Education in Wroclaw, Wroclaw, Poland; ^4^Faculty of Educational Studies, Adam Mickiewicz University, Poznań, Poland

**Keywords:** academic performance, child development, fine motor skills, gross motor skills, learning

## Abstract

Despite the general agreement that the interdisciplinary model of physical education (PE), based on the incorporation of core academic subjects into the PE curriculum, stimulates the holistic development of students, there is still a lack of methods for its implementation. Therefore, Eduball was created, i.e., a method that uses educational balls with printed letters, numbers, and other signs. Numerous studies have shown that children participating in activities with Eduballs can develop their physical fitness while simultaneously improving their academic performance, particularly in math and language, including some writing skills. However, little is known about the effects of Eduball on children’s graphomotor skills, which are key for the academic performance of students throughout the entire schooling process. Here, we investigate whether 6-month participation in PE with Eduball stimulates graphomotor skills in primary school students, such as drawing prehandwriting letter patterns on unlined or lined paper and rewriting text on unlined or lined paper. Our results show that the Eduball class (*N* = 28) significantly improved these skills compared to the control class (*N* = 26) participating in traditional PE. For example, students from the experimental group wrote with a lower pen pressure and better stability of the line, in contrast to those from the control group. Therefore, this study demonstrates that the Eduball method successfully supports teachers in developing graphomotor skills in children. More broadly, our findings make clear once again that there is the need to integrate physical and cognitive development in education, which can be achieved by using an interdisciplinary model of PE.

## Introduction

Cognitive and physical development are undoubtedly closely related and to some extent interdependent (Samuelsson and Carlsson, 2008; Vazou and Smiley-Oyen, 2014; Lindt and Miller, 2017). However, this relationship is not reflected in schools where cognitive and physical activity (PA) are often separated in time and space (Vazou et al., 2012). Students acquire key competencies for effective functioning in the modern world, i.e., writing and reading in one’s native language (EUR-Lex, 2006), by spending a large part of their school time sitting at a desk (Singer et al., 2006). Moreover, pressure to meet stringent requirements for academic achievements with an already crowded curriculum prioritizes core academic subjects, such as literacy and numeracy, over physical education (PE; Mahar et al., 2006; Zach et al., 2016). As a result, the time devoted to PE is still being reduced or its reduction is being considered (its instructional time already corresponds to slightly less than 10% of the total taught time at school, EACEA, 2013), and PE itself becomes only a short form of physical activity, focused on developing physical and health literacy and active lifestyles, and the prevention of chronic diseases and mental health disorders (World Health Organization, 2018), devoid of cognitive aspects (Cone et al., 2009; Tomporowski et al., 2015; Zach et al., 2016).

Research shows that such an approach is not beneficial. Quality physical education can impact not only physical and health literacy and movement proficiency but also social and cognitive skills and, consequently, influence academic outcomes (Ericsson, 2008; Ericsson and Karlsson, 2014; Kall et al., 2014; Marttinen et al., 2017; Mullender-Wijnsma et al., 2019; Norris et al., 2020; see also UNESCO, 2015 and World Health Organization, 2018). Moreover, integrating PA with teaching content in the classroom (classroom-based PA) may enhance children’s academic performance, e.g., has a positive effect on learning to read, write, and math (Marttinen et al., 2017; Watson et al., 2017; Norris et al., 2020). The same applies to incorporating core academic subjects into the PE curriculum, which is called the interdisciplinary model of PE (Zach et al., 2016; Marttinen et al., 2017). Through this teaching model, students gain essential kinesthetic learning experiences that enhance their ability to learn both movement and other subject areas through movement. The discipline’s unique ability to kinesthetically, cognitively, and affectively integrate content from other subject areas effectively addresses the needs of the whole child. Although the concept of an interdisciplinary model of PE is not new, there is still a lack of methods or tools for its implementation (Cone et al., 2009).

**Figure 1:**
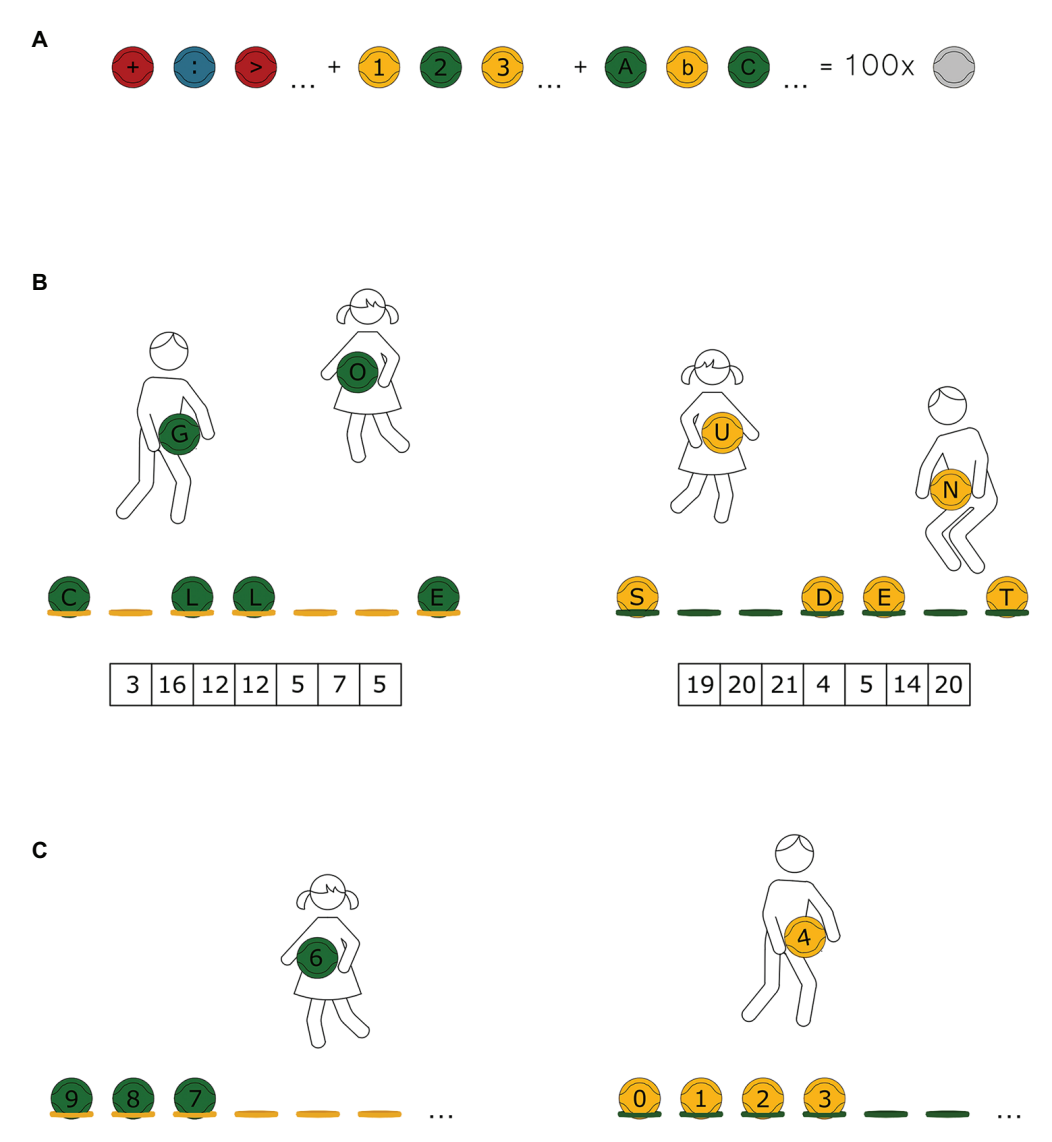
Eduball educational balls. **(A)** A set of Eduballs includes 100 balls used for team mini-games in five colors (blue, green, orange, red, and yellow) with painted (black) letters of the alphabet (upper and lower case), numbers from 1 to 9 and 0, symbols of mathematical operations, such as addition (+), subtraction (−), multiplication (^*^), division (:), greater than (>), and smaller than (<). **(B)** Example of math Eduball-game: “From 0 to 9.” The students, divided into two teams (yellow and green), freely move across the whole gym. At the educator’s signal, the pupils with the yellow balls need to line up as fast as they can from smallest to biggest number. The green team has to line up from largest to smallest number. **(C)** Example of language Eduball-game: “Enigma.” The learners, divided into two teams (yellow and green), obtain coded messages (numbers represent the order of letters in the alphabet). The pupil’s task is to decode the message and arrange the word from the letters on the balls.

Given the above considerations, in Poland, a method called “Eduball” has been developed over almost 2 decades. Currently, Eduball is included in the official national list of didactic aids for schools designated for use in teaching in preschool and primary schools and is recognized and approved by the Ministry of National Education of Poland and Polish Parliament’s Commission for Sport and is used in several 100 schools in Poland, as well as in other European countries, and in the United States (Cichy et al., 2020). In short, Eduball is based on an interdisciplinary model of teaching PE and combines physical activity and academic learning. The concept relies on the development and improvement of children’s academic performance through movement and play. The Eduball approach uses a didactic teaching aid in the form of educational balls (Eduballs) to integrate various subjects, such as language studies and mathematics, into PE (Rokita and Cichy, 2013). A set of Eduballs includes 100 balls for small team games in five colors (yellow, green, blue, red, and orange) with painted (black) letters of the alphabet (in upper and lower case), numbers (from 0 to 9), and signs for mathematical operations (e.g., addition, subtraction, multiplication, and division; Rokita and Cichy, 2013). There is no curriculum of Eduball-class. Eduball should naturally merge with school learning, and teachers can use a book with examples of Eduball-games (e.g., Rokita et al., 2017b). Ideally, educators should build their own solutions adjusting the activities to the presently studied material (for more details, see Cichy et al., 2020). Figure 1A demonstrates elements of the Eduball set. Figure 1B shows an example of an educational game with Eduball stimulating mathematical development, and Figure 1C – linguistic development (for more examples, see supplementary material of our recent, open access, Eduball-paper: Cichy et al., 2020).

Our previous studies (e.g., Rokita et al., 2017a, 2018; Cichy et al., 2020) have shown that children participating in activities with Eduball can develop their physical fitness, motor skills, and fundamental movement skills while simultaneously learning and improving their academic performance, particularly in math and language (for more details, see Cichy et al., 2020). Although we have shown that the use of Eduball improves some writing skills in children (e.g., the ability to write straight within the lines, Rokita et al., 2007), little is known about the effects of school-based PE with Eduball on children’s graphomotor skills. This context seems to be very important. Graphomotor skills, which involve the strength and control of finger muscles and incorporate important daily skills, such as handwriting and drawing, are necessary for the academic performance of students throughout the entire schooling process (Gallahue et al., 2011). Moreover, many studies show (e.g., Graham, 2010; Puranik and Lonigan, 2014) that students with good handwriting in their early years tend to succeed in school, while students lacking fluency in handwriting struggle in later years, which can affect their grades due to poorly and unreadable written compositions. However, teaching handwriting seems to be still challenging for schools and educators (Dinehart, 2015; Feng et al., 2017).

In its most basic form, handwriting is an exercise of fine motor control, and fine motor activities are considered to stimulate the prefrontal cortex (for details on the neural network involved in handwriting, see Longcamp et al., 2016; for neural correlates of handwriting, see Richards et al., 2011 and Berninger, 2019), which is an area of the brain that hosts elements of self-regulation and executive function (Diamond, 2000). Fine motor skills are characterized by small muscle movements that usually occur in the fingers (Gallahue et al., 2011) that interact with more proximal parts (wrist, forearm, upper arm, shoulder, and spine) to provide the stability needed for the fingers to function with skill (Erhardt and Meade, 2005). To become proficient writers in either handwriting, as well as keyboarding [handwriting and keyboarding are not antagonistic; each skill uses different motor and cognitive skills, but both situations involve motor learning; keyboarding builds upon the skills of handwriting; therefore, teaching handwriting is important in early grades, followed by keyboarding instruction in later elementary grades (Stevenson and Just, 2014); both teaching handwriting and keyboarding may be relevant to cognitive development (Alstad et al., 2015)], children must have graphomotor competence (Connelly et al., 2007). Improving graphomotor skills is usually linked to mastering the skill in the classroom by practising pen-and-paper exercises; however, practising manipulation tasks, such as building with blocks, weaving string, lacing beads, or cutting with scissors, also improves handwriting performance (Dinehart and Manfra, 2013). Moreover, a study by Westendorp et al. (2014) shows that the use of balls by children during classroom activities or physical education may also directly improve children’s manipulative and writing skills. Therefore, we hypothesize that such an interdisciplinary approach to PE as Eduball stimulates graphomotor skills by primary school students. In this brief research report, we test this hypothesis and show that Eduball is a method that effectively supports teachers in developing graphomotor skills in children and, more broadly, allows them to successfully implement an interdisciplinary model of PE in their work.

## Materials and Methods

### Participants

Fifty-four Polish students from first-grade classes (26 girls, age: 7–8, mean = 7.3, *SD* = 0.4) participated in the experiment. Classes were randomly assigned to the experimental and control groups. The experimental group included 28 children (16 girls), while the control group comprised 26 students (10 girls). Tests of cognitive and motor abilities were conducted (e.g., the 2HAND tests from the Vienna Test System, Schuhfried GmbH, Austria) to verify whether the control and experimental groups were homogeneous. No significant differences were found between the groups. In both groups, the regular classroom lessons were taught by the classroom teachers, and the PE classes were taught by PE specialists.

### Procedure

The study was approved by the Ethics Committee of the University School of Physical Education in Wroclaw, Poland, and all procedures and manipulations were carried out in accordance with the principles of the Helsinki Declaration. Written informed consent was obtained from the school headmaster, teachers and children’s parents or guardians.

The experiment lasted 6 months and was performed during the spring semester (January–June) in natural conditions (at school) using the technique of parallel groups (experimental and control). At the beginning and end of the semester, assessments of the students’ graphomotor skills were performed. A pretest was performed in January (Examination 1), followed by the PE program with Eduballs. The posttest was performed at the end of the experiment in June (Examination 2). The pretests and posttests were administered by specially trained researchers during the school’s daily timetable. The goals of the program for the experimental and control groups were based on the core curriculum of the Polish National Ministry of Education. The same curriculum was applied to both the experimental and control groups. The experimental factor in this study was a standard PE program integrated with literacy and numeracy content presented using Eduball.

The experimental group followed a PE program enhanced with Eduball twice a week at the sports hall. The Eduball activities lasted for approximately 30 min during each lesson. During the remaining time of each lesson, i.e., approximately 15 min, and the third obligatory PE hour, the teacher followed the standard PE curriculum in accordance with the school’s program (without Eduball). Thus, all the critical elements of the standard PE curriculum related to developing physical fitness and health education were maintained. However, Eduball allowed PA to be integrated with cognitive activity, and therefore an interdisciplinary model of PE to be implemented. The Eduball program consisted of 32 lessons. In each lesson, games and exercises from Eduball-pool of examples (Rokita et al., 2017b, 2018) were used. In Supplementary Material, we provide a detailed description of all games and exercises used, including a clarification of the goals and skills to be developed. In the experimental group, Eduball was used during classes for exercises, games, or play, and the PE activities were based on earlier prepared lesson plans integrated into language studies and mathematics exercises. The PE teacher cooperated with the experimenter and the classroom teacher to teach the lesson in accordance with a specially prepared plan that coincided with the classroom activities, the school’s cycle of weekly activities, and each day’s topic (see Supplementary Material for a sample scenario used during experiment; note that this example shows also how Eduball games and exercises were sometimes slightly modified depending on the topic of the day). The plans were prepared weekly by the experimenter and the PE teacher together after consultation with the classroom teacher. The experimenter observed each lesson and recorded what had been completed or had to be modified or covered during the next lesson.

The control group followed the standard PE program (three times per week for 45 min). The PE teachers conducted the PE program without Eduball in accordance with the aims and objectives of the school’s program for developing physical fitness and health education.

Graphomotor skills were assessed using a standardized Polish test called the Profile of Graphomotor Efficiency (Domagala and Mirecka, 2010) appropriate for children aged 7–13. The test consisted of the following four diagnostic trials: (1) drawing prehandwriting letter patterns on unlined paper, (2) drawing prehandwriting letter patterns on lined paper, (3) rewriting text on unlined paper, and (4) rewriting text on lined paper. The products of the graphomotor activities for each trial were evaluated in terms of 13 categories, such as line, letters and prehandwriting letter patterns, letters in a word or prehandwriting letter patterns in a structure, record of the text or a prehandwriting letter pattern, arrangement of text, and organization of the page. All categories were evaluated by scores ranging from 0 to 3 points (zero points were given for the smallest deviation from the norm, and three points corresponded to the largest deviation). Therefore, in each trial, the participant could receive a maximum of 39 points and 156 points in the entire study. Higher points indicated poorer handwriting performance.

### Data Analysis

The main dependent variables were mean scores for each trial of the graphomotor test, calculated separately for each group (Experimental and Control) and test (Examination 1 and 2). First, using the one-sample Kolmogorov-Smirnov test, we confirmed the normality of the distribution of all variables (all *p* between 0.06 and 0.20). Then, we ran an independent samples *t*-test to compare the differences in the results of the pretest between two independent comparison groups (Experimental vs. Control). Finally, paired samples *t*-test was run to compare mean scores from pretest and posttest (Examination 1 vs. Examination 2), and therefore between two dependent comparison groups. The adopted level of significance was *α* = 0.05. An effect size was calculated using Cohen’s *d* and interpreted as: <0.2 – very small or no effect, 0.2–0.5 – small, 0.5–0.8 – medium, 0.8–1.2 – large, and >1.2 – very large or huge (Sawilowsky, 2009). All statistical analyses were carried out using IBM® SPSS Statistics® for Mac Version 26.0 (IBM Corp., Armonk, NY, United States); however, to calculate effect sizes, we used the Effect Size Calculator for T-Test® (Social Science Statistics, https://www.socscistatistics.com, 19-09-2020).

## Results

**Figure 2:**
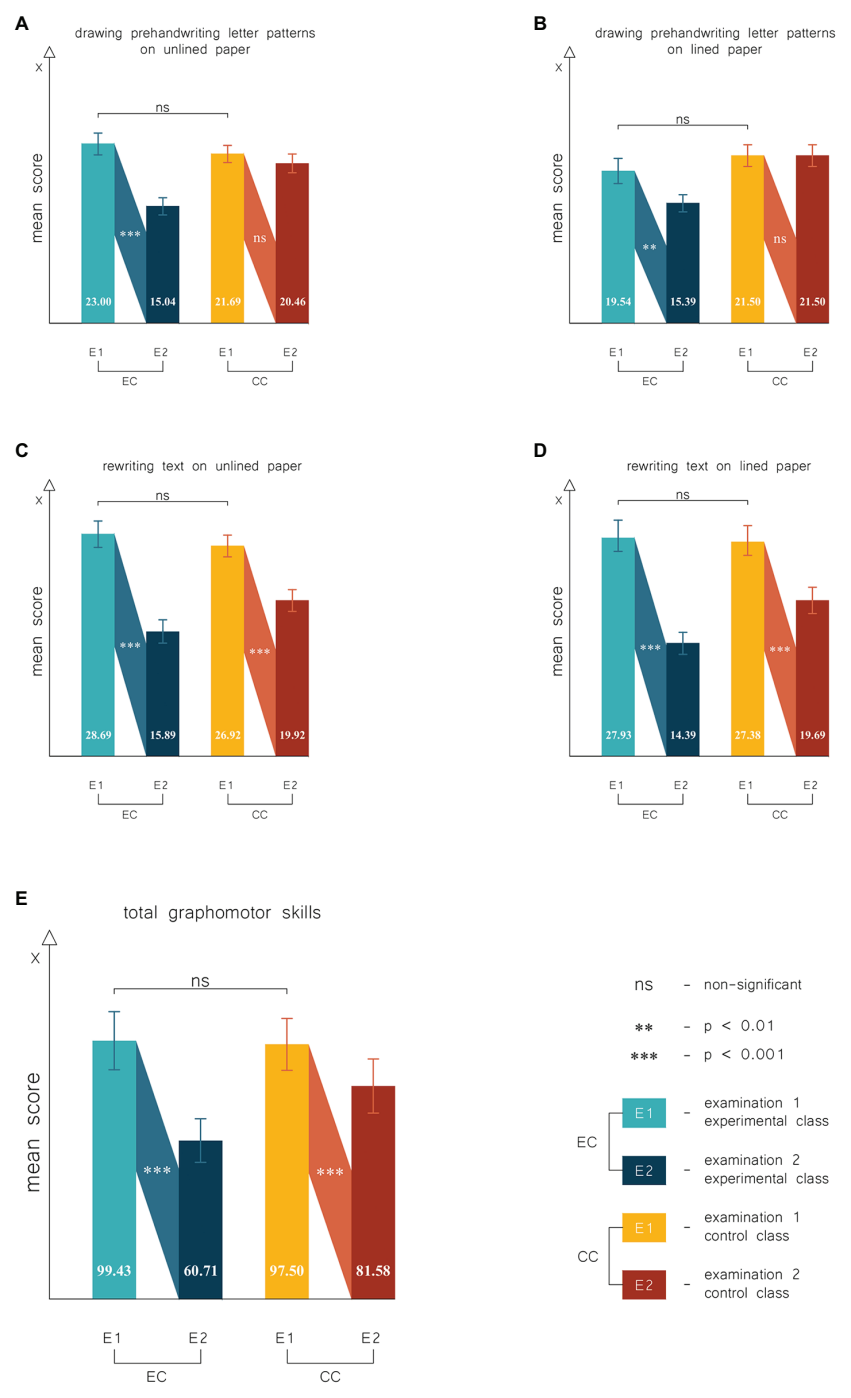
Results of the experiment in terms of graphomotor skills. **(A)** Results of trial: drawing prehandwriting letter patterns on unlined paper. **(B)** Results of trial: drawing prehandwriting letter patterns on lined paper. **(C)** Results of trial: rewriting text on unlined paper. **(D)** Results of trial: rewriting text on lined paper. **(E)** Results of the experiment in terms of the total graphomotor skills (the sum of four trials). Higher points indicated poorer handwriting performance. Error bars depict SEs of the means. EC, experimental class; CC, control class; Examination 1, pretest carried out at the beginning of the spring semester (in January); and Examination 2, posttest carried out at the end of the school year (in June).

At the beginning of the experiment, there were no significant differences between the groups in terms of graphomotor skills, both taking into account the results of individual trials of the test (Figures 2A–D), as well as the overall test result (Figure 2E). The experimental factor, however, changed this situation, and after a 6-month experiment, the groups began to differ in terms of graphomotor competences, which was beneficial for the experimental group.

Eduball significantly improved the graphomotor skills of students examined in the first two trials. In the trial of “Drawing prehandwriting letter patterns on unlined paper” (Figure 2A), their score decreased by an average of 7.96 points (*SD* = 7.90, *t* = 5.34, *p* < 0.001, *d* = 1.22), and in the trail of “Drawing prehandwriting letter patterns on lined paper” (Figure 2B), by an average of 4.14 points (*SD* = 6.98, *t* = 3.14, *p* < 0.01, *d* = 0.57). No significant progress for these trials was observed in the control group (both *p* > 0.05; Figures 2A,B).

In both groups, we found an improvement in graphomotor performance in the range of two subsequent trials, but the effect was greater in the Eduball group. In the trial “Rewriting text on unlined paper” (Figure 2C), the students from the experimental group improved their result by an average of 13.07 points (*SD* = 9.23, *t* = 7.49, *p* < 0.001, *d* = 1.74), and those from the control group improved their result only by an average of 7.00 points (*SD* = 6.05, *t* = 5.90, *p* < 0.001, *d* = 0.98). In the last trial, “Rewriting text on lined paper” (Figure 2D), Eduball-students improved their score by an average of 13.56 points (*SD* = 7.73, *t* = 9.26, *p* < 0.001, *d* = 1.58), and no-Eduball improved their score only by an average of 7.69 points (*SD* = 6.65, *t* = 5.90, *p* < 0.001, *d* = 0.96).

When analyzing the cumulative test score, we also found that students from both groups developed their graphomotor skills, but for the students participating in Eduball activities, this effect was much greater. As can be seen in Figure 2E, the students from the no-Eduball group improved their overall result only by an average of 15.92 points (*SD* = 17.69, *t* = 4.59, *p* < 0.001, *d* = 0.61), while those from the Eduball group improved their overall score by an average of 38.71 points (*SD* = 25.71, *t* = 7.97, *p* < 0.001, *d* = 1.47).

## Discussion

Our study shows that 6 months of participation in PE with Eduball stimulates such graphomotor skills by primary school students as drawing prehandwriting letter patterns on unlined or lined paper and rewriting text on unlined or lined paper. This effect may be because physical activity with Eduball (e.g., games, playing, exercises, and various tasks, such as practice ball passes and grips, bouncing and throwing, as well as returning and receiving) engages the whole body and many muscle groups simultaneously and may strengthen the muscles, relieve tension in the hand, and develop fine and gross motor skills (Westendorp et al., 2014) and, as a result, improve drawing and writing performance (Bara and Gentaz, 2011). However, the observed improvement in graphomotor functioning does not seem to be just an effect of varied and whole-body physical activity. Eduball lessons are integrated with the content that children learn in the classroom. Thus, the greater progress in graphomotor skills in the experimental group compared to the control group may be caused by combining movement with cognitive activities. For example, placing letters on the balls and merging academic content with PA may result in better memorization of letters and language rules, and some evidence suggests that letter memorization and learning handwriting are closely related and interact in a way with each other (Mangen and Velay, 2010). Having fun can also have some impact here. Most children enjoy playing with balls because ball activities can present a wide range of varied and exciting experiences that are fun and challenging (Wheeler and Spilker, 1991). Fun is an important factor in the learning process, positively influencing both cognitive (Diamond, 2012) and physical development (Burns et al., 2017).

Our findings are in line with studies that emphasize the need for significant changes in the curriculum and school program to integrate PE with didactic content to positively influence the academic experiences and achievements of children (Macdonald, 2011; Zach et al., 2016; Marttinen et al., 2017). However, many studies have shown that responding to this need is difficult (for review, see Horvat et al., 2019; Pangrazi and Beighle, 2019; Cecchini and Carriedo, 2020; Capel et al., 2021). First, physical educators, parents, and principals express concerns regarding whether an interdisciplinary approach to PE enables the attainment of physical education aims and objectives. Second, major curricular changes are formally difficult. Finally, no single model that describes all the ways interdisciplinary instruction can be delivered exists; therefore, an interdisciplinary approach to PE is a challenge for teachers that may discourage them (Cone et al., 2009). Eduball is a method that overcomes such difficulties to a large extent. In the case of Eduball, there is no risk of deterioration in physical fitness. Previous Eduball studies (e.g., Rokita et al., 2017a) have shown no negative effects of additional academic instructions in PE on physical fitness and motor performance. Furthermore, the PE program with Eduballs does not require drastic changes in the core curriculum, but teachers must only be willing to integrate academic instructions and activities into PE. Of course, PE teachers need to be prepared to integrate core subjects into PE (Zach et al., 2016; Marttinen et al., 2017), and mastering new routines takes some time (Fullan, 2007); however, the Eduball method assumes that they are assisted by other teachers. In our project, PE was taught by a PE teacher who collaborated with the classroom teacher while preparing lesson plans. A previous study (Zach et al., 2016) suggests that such collaboration between PE teachers and other class teachers most likely has a positive effect on student performance. Similarly, one of our earlier Eduball studies (Rokita et al., 2017a) confirmed that collaboration between specialized PE (who use the Eduball method) and classroom teachers has a positive influence on children’s academic (reading, writing, and math) and motor skills. This cooperation may also have a different direction. Instead of integrating academic core content into physical education (as is the case with the interdisciplinary model of PE), physical activity can be integrated into classroom academic lessons or PA breaks (i.e., as in the classroom-based PA); however, this process may involve challenges, and training may be required (Stylianou et al., 2016). Further research is needed on this issue (and as writing development continues beyond primary education, see Bazerman et al., 2017, 2018, older students should also be involved). It seems that using Eduball in the classroom would also require a miniaturization of these educational balls. We are currently developing a prototype of this type of mini-Eduball (the size of a tennis ball), which, by integrating cognitive activity with fine motor tasks, can offer even greater benefits than “big” Eduballs, as neural correlates of fine motor skills are much more closely related to language or numeracy neural circuits, compared to neural underpinnings of gross motor abilities (Bidula et al., 2017; Klichowski and Kroliczak, 2017, 2020; Przybylski and Kroliczak, 2017; Styrkowiec et al., 2019; Klichowski et al., 2020; Kroliczak et al., 2020; for the most recent evidences, see Kroliczak et al., 2021).

Overall, Eduball is an effective method of an interdisciplinary model of PE by which primary school students can improve their physical condition and fitness while simultaneously developing graphomotor skills. Moreover, Eduball is a relatively easy-to-use approach, and its implementation in education does not require major changes to the curriculum. Future research should focus on strategies to include education balls not only in PE but also in classroom lessons so that Eduball is used by classroom teachers in addition to PE teachers. Miniaturization of Eduballs could be one solution to allow this and at the same time to increase the effects of the Eduball method even more.

## Data Availability Statement

The raw data supporting the conclusions of this article will be made available by the authors, without undue reservation, to any qualified researcher.

## Ethics Statement

The studies involving human participants were reviewed and approved by Ethics Committee of the University School of Physical Education in Wroclaw, Poland. Written informed consent to participate in this study was provided by the participants’ legal guardian/next of kin.

## Author Contributions

This project was conceptualized by SW and AR. Data were collected by SW and DP, analyzed by MK, and interpreted by SW, IC, AM, DP, MK, and AR. The manuscript was written by SW, MK, IC, and AR. Figures were prepared by AK. All authors contributed to the article and approved the submitted version.

### Conflict of Interest

The authors declare that the research was conducted in the absence of any commercial or financial relationships that could be construed as a potential conflict of interest.
