# Supplementary material for: Physical Activity With Eduball Stimulates Graphomotor Skills in Primary School Students
Source: Front Psychol. 2021 Mar 4;12:614138. doi: 10.3389/fpsyg.2021.614138 (PMC7969879; doi:10.3389/fpsyg.2021.614138)
Supplement: Supplementary file 1 [file Data_Sheet_1.pdf]

Supplementary Material

1. Games and Exercises used during Experiment (based on Rokita et al., 2017; 2018)

1.1. *WHAT COLOR IS IT?*

- Objective: to develop logical thinking and improve quick reaction time, speed and nimbleness
- Number of participants: any number
- Area: sports hall
- Description: Each student has a green or yellow ball and moves freely about the gymnasium playing with it. At the teacher's signal, the students exchange their balls while naming the color of their ball (e.g., "this is green", "this is yellow", "this is red", "this is blue", "this is orange").
- Variation: the students use full sentences in English (e.g., "This is a blue ball", "This is a red ball", "This is a green ball", "This is a yellow ball", "This is an orange ball").

1.2. *PAIR UP*

- Objective: to practice the distinction between colors, letters and digits and improve certain motor skills
- Number of participants: any number
- Area: sports hall
- Description: Each student has a green or yellow ball. They are galloping across the gymnasium when, at the teacher's signal, they have to pair up in indicated combinations of colors, e.g., yellow and yellow, green and green, red and blue, yellow and green etc.
- Variation I: At the teacher's signal, students have to pair up in indicated combinations of letters and colors, e.g., vowels and consonants in the same colors or vowels and consonants in separate colors etc.
- Variation II: At the teacher's signal, students have to pair up in indicated combinations of numbers and colors, e.g., even and odd numbers in the same colors or even and odd number in separate colors.

1.3. *LETTERS*

- Objective: to practice the order and pronunciation of the letters of the alphabet and improve certain motor abilities and physical skills
- Number of participants: any number
- Area: sports hall
- Description: The students move across the gymnasium playing with yellow and green balls in a manner indicated by the teacher, e.g., rolling or dribbling. At the teacher's signal, they stop and say the letter they have on their ball aloud, e.g., D – a dog, H – a house etc. After that, they exchange balls.

1.4. *THE DRILL*

- Objective: to practice the distinction between vowels and consonants, teach organizational and ordering activities, and improve certain motor skills

- Number of participants: any number
- Area: sports hall
- Description: The students have green and yellow balls. The teacher indicates a line where students should line up depending on the letter they have on their ball, e.g., vowels should line up on the sidelines and consonants on the end lines of the volleyball court. At the teacher's signal, the students move freely around and exchange their balls with any person they meet on their way on the court. At the signal "DRILL", the students should get back to their lines as soon as possible. The teacher checks the correctness of the line-up and if the vowels and consonants have been separated accordingly.
- Variation: The teacher changes the place and the manner in which the students should regroup, e.g., vowels line up and the consonants should sit cross-legged at the center of the circle.

1.5. ***VOWELS AND CONSONANTS***

- Objective: to develop language skills, improve running speed, and practice certain motor skills
- Number of participants: any number
- Area: sports hall
- Description: Each student has a ball and moves about randomly dropping and catching the ball before it bounces twice. At the teacher's signal, the students pair up (according to the letters on their balls). Students who have consonants find students with vowels to form syllables, e.g., "in", "on", "at". At the end of the game, the students read the syllables they have formed.

1.6. ***SYLLABLES***

- Objective: to teach one-syllable and two-syllable word formation and improve certain motor skills
- Number of participants: any numer
- Area: sports hall
- Description: Each student has a green or yellow ball and moves about randomly throwing/ bouncing/catching the ball with both hands. At the teacher's signal, the students form two-and three-letter syllables, e.g., "win", "la", "ter", "zy", "wa". At the teacher's signal, they run together across the gymnasium, and at the second signal, they look for other students with whom to form two-syllable words and read them aloud, e.g., winter, lazy. The students who have not found a pair have to say a word that contains their syllable.
- Variation: Depending on how advanced the group is, three- to four-syllable words can be created.

1.7. ***WHO IS CALLED?***

- Objective: to improve knowledge of the letters, enrich vocabulary and improve certain motor skills (speed)
- Number of participants: any number
- Area: sports hall
- Description: The students stand in a circle and hold a green or yellow ball. In the center of the circle, there is a teacher who calls a two-letter word, e.g., "at". The students who have balls with the letters "A" and "T" run around the circle and return to their place. The winner is the fastest student. After each word or few words, the students should exchange balls (e.g., passing the ball two places to the right/left) to follow the principle of equal opportunity rules.

- Variation: the teacher calls three-, four-, five-letter words, e.g., “cat”, “auto”, or “chair”.

1.8. *WORDS*

- Objective: to enrich vocabulary and improve certain fundamental movement (locomotor and object-control) skills
- Number of participants: any number
- Area: sports hall
- Description: Each student has a green or yellow ball. They perform various locomotor skills, e.g., galloping or jumping around the gymnasium. At the teacher’s signal, the students stop and say a word or words that begin with the letter on their ball, e.g., “APPLE”, “KEY”, “RIVER”. Then, the students should perform object-control skills, e.g., bounce or roll a ball as many times as is the number of the letters in the word, e.g., when the student says “APPLE”, he/she should bounce the ball 5 times.

1.9. *LETTER TAG*

- Objective: to develop the skill of forming words beginning with a certain letter and improve certain motor abilities and physical skills
- Number of participants: any number
- Area: sports hall
- Description: Each student has a yellow or green ball. Students who have a ball with the letter, e.g., “B”, are the chasers. Their task is to tag the rest of the students. A participant who has been tagged by the chaser can gain protection if they say a word that begins with the letter “B”, e.g., “BALL”, “BOX”. They only have 5 seconds to do so and cannot repeat what has already been said. When someone says an incorrect word, repeats a word or takes too much time, they become the chaser.
- Variation: The students can also gain protection by saying a word that only contains the letter “B”, e.g., “RABBIT”, or ends with it, e.g., “CLUB”.

1.10. *WORD TAG*

- Objective: to develop the skill of forming words beginning with a certain letter and improve certain motor abilities and physical skills
- Number of participants: any number
- Area: sports hall
- Description: The students have the green or yellow balls. Two students have the red or blue balls, and they are the chasers. When the chasers tag someone, they have to stop with their legs apart and their arms holding a ball showing a letter on the ball, e.g., “S”. They may be freed by another student by saying a word that begins with the letter on the ball, e.g., “SCHOOL” and crawling between their legs.

1.11. *A AS IN ABC*

- Objective: to develop the skill of forming words beginning with a certain letter, improve the ability to work in a team, and improve certain motor abilities and physical skills
- Number of participants: any number
- Area: sports hall
- Description: The students are divided into two teams (yellow and green team). Each team gets a ball with the letter, e.g., “A”, which they place on a rubber ring at the starting line. The students’ task is to come up with as many words as they can beginning with the letter “A”. The yellow team uses the green balls and the green team uses the yellow balls. The students must

cooperate to transport the balls to their starting lines. They cannot move with the ball in their hands. They need to pass the ball to each other while transporting it to their line. They have 5 minutes to complete the task. After this time, the teacher checks the correctness of the words. The teacher can award 1 point for each word or one point for each letter in a word. The winning team is the team that scored the most points.

1.12. *MY FRIEND IS…*

- Objective: to improve the ability to form certain parts of speech, enrich vocabulary, and improve certain motor skills
- Number of participants: any number
- Area: sports hall
- Description: The students sit in the center circle of the basketball court. Each student has a yellow or green ball. The teacher asks a question: "What is your friend like?" The students have to describe their friend using a word that begins with the letter they have on their ball. When a student says "My friend is helpful", each student who agrees with the statement should perform a motor task as many times as is the number of the letters in the given word, so in this case, seven squats, seven jumps, etc.
- Variation I: The students have to answer the question "What do you like?" They have to respond with a noun word, e.g., "I like e.g., music".
- Variation II: The students have to answer the question "What do you like to do?" They have to respond with a verb, e.g., "I like swimming".

1.13. *ANTONYMS*

- Objective: to enrich vocabulary (antonyms), develop creativity and abstract thinking, and improve certain motor skills
- Number of participants: any number
- Area: sports hall
- Description: The students are divided into four groups, and they form the lines. Each team draws cards with words prepared by the teacher. The first student whispers a message (the first letter of the word) to the ear of the next person in the line, and so on, until the last student stands up and runs to find a letter. When the word is formed, they have to tell the antonym of it and together form it next to the word, e.g., "slow" – "fast", "sad" – "happy", "hot" – "cold", "week'"" – "strong". The teacher checks the correctness of the words. All the groups form sentences with their antonyms.

1.14. *WORD FORMATION*

- Objective: to improve language skills and improve locomotor skills
- Number of participants: any even number
- Area: sports hall
- Description: The students are divided into three teams. The balls are in the zone in a designated place in the gym. The teams should form and write down as many words in a certain thematic category, e.g., nature. After having formed each word, they write it down and roll the balls back to the zone. Then, all the members of the team have to run around the cone while holding hands to score 1 point. After that, they can create the next word etc. The winning team is the team with the most words formed within five minutes.

1.15. *BRAINSTORMING*

- Objective: to enrich vocabulary in a certain thematic category (e.g., spring), develop creativity and abstract thinking, and improve certain motor skills
- Number of participants: any number
- Area: sports hall
- Description: Each student has a yellow or green ball. They move across the gym in various directions. At the teacher's signal, the students run up to one of the boards, where they write down a word they associate with the given season. The word has to start with the letter they have on their ball. After having written down the word, the students exchange balls and continue the game. At the end of the game, the students sit down in a circle with the teacher and read the collected words.

1.16. *PASSWORD*

- Objective: to improve the ability to form words, expand vocabulary in a certain thematic category (e.g., holidays), develop the ability to work in a team, improve certain motor skills
- Number of participants: any number
- Area: sports hall
- Description: The students are divided into two teams. The first task for each team is to form the word "holidays" using the balls. Next, the students have to form 8 words that they associate with holidays. Each word should contain one letter from the theme H-O-L-I-D-A-Y-S. After they have formed a word, all the students of the team should perform a motor task (e.g., A-skip or C-skip) as many times as is the number of the letters in the word they have formed. After they have completed the task, the students read their words and relay the number of letters, speech sounds and syllables in each word.

1.17. *RIDDLES*

- Objective: to develop creativity and logical thinking, improve the ability to work in a team, and practice certain motor skills
- Number of participants: any number
- Area: sports hall
- Description: The students are divided into teams of 4-6. Their task is to find a solution to the riddle and to form the answer using balls. At the end of the game, the students share their solutions with the others.

1.18. *CHARADES*

- Objective: to develop creativity and abstract thinking, improve the ability to work in a team and practice certain motor skills
- Number of participants: any even number
- Area: sports hall
- Description: The students are divided into two teams (yellow and green). The teacher hands out cards to each team with words in specific categories, e.g., household goods: oven, fridge, toaster, dishwasher, microwave. The cards are marked with the number of the letters in the word that is the point value for the word, e.g., dishwasher = 10 points. One student in each team shows by speechless gesticulating what the word is. The teams have to guess what the word is and form the name on the rubber rings. Then they are awarded points. The presenting student can choose the word by the number of the letters. After each word, there is a change of presenter. The game lasts 5-10 minutes. At the end of the game, the students sum up the points for each word, and the winning team is announced.

1.19. ***FIND AND SIGN IT***

- Objective: to practice knowledge acquired in science education, improve the ability to work in a team, develop orientation in space and time
- Number of participants: any even number
- Area: sports hall
- Description: The students are divided into four teams. Each team gets a picture, e.g., of a spring flower. Several notes with the written names of the items in the pictures are hidden under training cones in different spots in the gymnasium. The students' task is to find the relevant description under the cone on the pitch and form the name of what is on the picture out of Eduballs.

1.20. ***CROSSWORD***

- Objective: to practice knowledge in interdisciplinary educational areas and improve the ability to work in a team
- Number of participants: any even number
- Area: sports hall
- Description: The students are divided into two teams (yellow and green). The students line up at the starting line. Their task is to solve a crossword and form its solution out of balls. First, they have to solve the crossword at the halfway point. The students can reach the balls in pairs in a manner indicated by the teacher (run, jump, side step, A-skip, etc.). Each pair can answer only one question and write it down. After the crossword has been solved, all the students have to form the solution out of balls at the starting line. At the end of the game, the teacher awards points for speed and the correctness of the completed task.

1.21. ***NUMBERS***

- Objective: to learn about number names, to practice counting, and to practice certain motor skills
- Number of participants: any number
- Area: sports hall
- Description: The balls are strewn across one half of the gymnasium. The teacher calls a number and the students pair up. Their task is to find a ball with the number and perform a special task that has been assigned to each number and repeat it according to the number, e.g., 360° turns, jumps, squats.

1.22. ***BALL HUNTERS***

- Objective: to learn the counting sequence, count to tell the number of objects, improve social interactions, and improve certain motor skills
- Number of participants: any number
- Area: sports hall
- Description: The balls are strewn across one half of the gymnasium. The students sit cross-legged at the center circle. At the teacher's signal, they stand up and individually run to collect as many balls as they can hold. The students get 60 seconds to come back to the center circle holding as many balls as possible. The teacher asks the students to count the number of balls.
- Variation: the students work in pairs, trios and multiplayer groups.

1.23. ***CORNERS***

- Objective: to classify objects (set, subsets); describe their similarities, differences, etc.; and improve certain motor skills
- Number of participants: any number
- Area: sports hall
- Description: The students sit cross-legged at the center circle, and the balls are placed inside the circle. The students describe the balls and discuss the common features of a set of balls, e.g., subsets by colors, numbers, letters, characters, or shape. Then, they toss or roll all of the balls around the gymnasium. At the teacher's signal, the students group the balls into five places by color, e.g., in the corners of the gym and the central circle.
- Variation: The students can regroup the balls inside the color sets, e.g., odd and even numbers, four mathematical operations (addition, subtraction, multiplication, and division).

1.24. ***FROM 0 TO 9***

- Objective: to practice the order of digits in an increasing and decreasing number sequence and improve certain motor skills
- Number of participants: any even number
- Area: sports hall
- Description: The students are divided into two teams (yellow and green team). The students freely move across the whole pitch, pretending to be a bird, e.g., stork or eagle etc. At the teacher's signal, the students with the yellow balls need to line up as fast as they can from smallest to biggest number. The green team has to line up from biggest to smallest number.

1.25. ***EVEN AND ODD NUMBERS***

- Objective: to practice the distinction between even and odd digits and improve certain motor skills
- Number of participants: any number
- Area: sports hall
- Description: The balls are strewn across the whole gymnasium. The students stand at the center circle. The teacher indicates places for even and odd numbers in the corners of the gymnasium. The students move freely across the gymnasium. At the teacher's signal, they should put the balls into the indicated places according to previous instructions.
- Variation: Each student has a green or yellow ball. The teacher indicates bases for even and odd digits in the corners of the gymnasium. The students move across the gym in a manner indicated by the teacher. At the teacher's signal, the students have to put their ball into the right corner according to the color of the ball and the digit on it. After they have completed the task, the students exchange balls.

1.26. ***MATH RACES***

- Objective: to improve number recognition, solve problems involving the four mathematical operations (addition, subtraction, multiplication and division), and improve locomotor and object-control skills
- Number of participants: any number
- Area: sports hall
- Description: The students sit cross-legged in two teams on the line (yellow and green team) with balls with the numbers from 0 to 9 (depending on the number of the participants). In each team, the students count off the numbers in the order, e.g., from 1 to 9. At the teacher's signal, the first students run to find a ball with their number, and after their return, the next

person runs, etc. When all the balls are found, the teacher calls a number, e.g., "7", and the students who have balls with the number "7" stand up and run around the designated point. The teacher chooses the method of moving, e.g., running, tossing a ball, or dribbling. After each race, the teacher can award points for the teams: 2 points for a winner, and 1 point for the other student. The winner is the team that scored the most points.

- Variation I: The teacher shows and says a mathematical equation, e.g., 1+2=?. The students whose number on their ball is the result of the equation, e.g., "3", perform the task. In the case of two-digit results of mathematical operations, e.g., 6+7=13, two students perform the task in preserved order "1" and "3". After a few races, the students should regroup and exchange positions and balls, in accordance with the principle of equal opportunity.
- Variation II: When the teacher shows an equation, e.g., "1+2=3", the students with all the numbers, "1", "2" and "3", perform the task.
- Variation III: When the teacher shows an equation, e.g., "2+5=7", the students whose number on the ball is greater and less by 1 number than the results perform the task, e.g., 6 and 8.
- Variation IV: The difficulty of the equations is adapted to the age and skills of the students. The teacher can show mathematical operations involving subtraction, multiplication and division, etc.

1.27. *NUMBER TAG*

- Objective: to get familiar with the digit "5", learn mathematical operations with the number "5", develop orientation in space and time, and improve speed
- Number of participants: any number
- Area: sports hall
- Description: Each student has a ball in any color. The student who has the ball with the number "5" is the chaser. When the chaser tags a student, they exchange their balls and the tagged person becomes the chaser.
- Variation I: All the students have green or yellow balls. The student who has the ball with the number "5" on it is the chaser. A person who gets tagged by the chaser can gain protection if they say what the sum of their number and the number "5" is. They only have five seconds to do so. When someone says an incorrect sum or takes too much time, they become the chaser.
- Variation II: A person who gets tagged by the chaser can gain protection if they say what the sum of the number indicated by a teacher and the number "5" is.

1.28. *COMPARE SETS*

- Objective: to practice the use of mathematical signs (<, >, =) and mathematical operations, develop the ability to work in a team, and improve certain motor skills.
- Number of participants: any number
- Area: sports hall
- Description: the students are divided into five teams. Two hoops and a rubber ring between these hoops are assigned to each group. Eduballs are strewn across the pitch. The first task of each team is to place green and yellow balls into the hoops within 30 seconds. The next task is to count how many balls there are in each set and to place a ball with a sign (<, =, >) accordingly on the rubber ring. After the task has been completed, the whole team has to run around their sets three times as fast as they can and return to their starting/finishing position. After that, the teacher checks the correctness of the comparisons.
- Variation: a more difficult task would be to sum up the numbers on the balls in the hoops and placing the relevant sign (<, >, =) according to the sums, not the number of the balls.

1.29. ***SUNSHINE***

- Objective: to practice calculations, develop spatial abilities, and improve running speed
- Number of participants: any even number
- Area: sports hall
- Description: The students are divided into two teams. The teacher places a ball with the number "9" on a rubber ring for each team. The student's task is to form as many mathematical equations involving addition or subtraction, as they can resulting "9". The balls are spread on half of the gymnasium. The students work together to use all the balls to form the equations. They have 5 minutes to complete the task. Then, the teacher checks the correctness of the equations. The teacher can award 1 point for each equation. The winning team is the team that formed the most equations.

1.30. ***WORD PROBLEMS***

- Objective: to practice simple word problems, develop the ability to work in a team, and improve certain motor skills
- Number of participants: any even number
- Area: sports hall
- Description: The students are divided into two teams. The balls are strewn across the whole gymnasium. The students' task is to solve the word problem and to form with the balls the entire equation. The students move in pairs (each pair can only carry one ball). After they have formed the operation and found the solution, the students perform a motor task together, e.g., at the center circle, they all hold hands and do squats together; the number of squats depends on the solution, e.g., 15.

1.31. ***EDUBALL OBSTACLE COURSE***

- Objective: to practice mathematics and native language skills and improve nimbleness, speed and coordinated movement
- Number of participants: any even number
- Area: sports hall
- Description: The students are divided into four teams. Each student has a yellow or green ball. The students' task is to get across the obstacle course as fast as they can and to complete all the tasks at the stations marked with flags. At the first station, the students write down an equation with the number on their ball; then, they complete a part of the obstacle course: going through a hoop, running around a bench, slaloming between training cones and jumping over poles. At the second station, the students write down a word that includes as many letters as is the number on their ball and complete the next part of the obstacle course: going through a hoop, slaloming between training cones, two-legged jumping through a hoop. At the third station, the students write down a word beginning with the letter on their ball and move on to the next part of the obstacle course: going through a hoop, two-legged side jumping through an agility ladder, balancing over a bench turned upside down, one-legged jumping through a hoop. At the fourth station, the students fill in the blank in a mathematical equation and complete the last part of the obstacle course: going through a hoop, rolling sideways over a mat, going through a hoop and moving underneath poles.

1.32. ***BRAINBALL CREATIONS***

- Objective: to develop creativity and abstract thinking, improve the ability to work in a team and practice certain motor skills
- Number of participants: any even number

- Area: sports hall
- Description: The students are divided into four teams. They gather in four corners of the gymnasium. The balls are spread in the center circle of the basketball court. The students have to form any creation using of the specific features of the Eduballs (their colors, letters, numbers, mathematical signs, etc.). To move the necessary balls to their corners, the students need to cooperate. They may pass the ball from one to another, but they cannot move while carrying them. Only one ball at a time can be transported. They have five minutes to complete the task. At the end of the game, each team presents their work to the other teams, which try to determine what the creations are, e.g., clock, calculator, phone, license plate.

2. Sample Scenario used during Experiment

School's cycle of weekly activities: Discovering the truth about ourselves and others

Topic of the day: Characteristics of a good friend

**Objectives**	**Description (based on Rokita et al., 2017)**	
To enrich vocabulary (adjectives), and improve object-control skills (dribbling, catching, etc.)	The students stayed in a circle in the middle of the gymnasium. Each student had a yellow or green ball. The teacher asked, “What are the traits of a good friend?” The students had to say a word that began with the letter they had on their ball. For instance, when a student said “helpful”, each student who agreed with the statement had to perform a motor task as many times as the number of the letters in the given word, e.g., 7 dribbles.	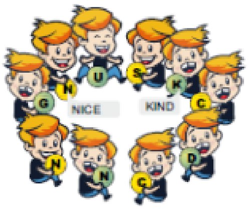
To develop the skill of forming words, cooperation in a group and improve locomotor skills	The students are divided into yellow and green teams. Each team gets a ball with the letter “a”, which they place on a rubber ring on the starting line. The students' task is to list and form as many words as they can beginning with the letter “a”. The yellow team uses the green balls, and the green team uses the yellow balls. The teams have 5-10 minutes to complete the task. Then, the teacher checks their accuracy. The teacher can assign a score of 1 point to each word or one point to each letter in a word. The winning team is the team that scored the most points.	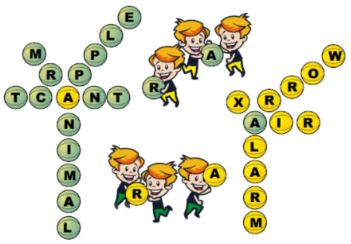
To practice simple word problems, cooperation in a group, and improve certain motor skills	The students work in the same teams. The students' task is to solve a word problem, such as “In the classroom, there were 6 girls and 9 boys. How many children were in the classroom?”, and form the entire equation with the balls. The students move in pairs (each pair can only carry one ball). After forming the operation and finding the solution, the students perform a motor task together, e.g., they all hold hands and perform squats together; the number of squats depends on the solution, e.g., 15.	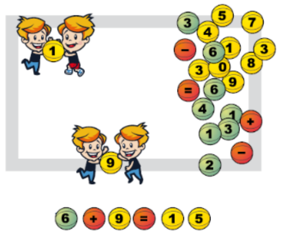

To practice numbers, improve cooperation in a group, and improve body coordination skills	Eduballs are spread around the gymnasium. Each team is a vacuum cleaner and collects as many balls as possible. The first person can collect a ball and pass it to the next student, etc., to the last person in the team. When all balls are collected, the teams count how many balls they have. The winner is the team with the most balls.	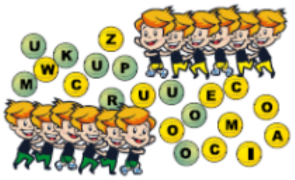

References

Rokita, A., Cichy, I., Wawrzyniak, S., and Korbecki, M. (2017). Eduball games and sports. A guide for primary school teachers and cooperating physical education teachers carrying out the “Little Champion” program. Wroclaw, Poland: Ministry of Sport and Tourism, Marshal Office of the Lower Silesia Region, and School Sports Association “Dolny Slask”.

Rokita, A., Wawrzyniak, S., and Cichy, I. (2018). Learning by playing! 100 games and exercises of Eduballs. Wroclaw, Poland: AWF.
